# Acupuncture as an independent or adjuvant therapy to standard management for menopausal insomnia: A systematic review and meta-analysis

**DOI:** 10.1371/journal.pone.0318562

**Published:** 2025-02-06

**Authors:** Xiaoni Zhang, Chengyong Liu, Shan Qin, Chaoming Chen, Xiaoqiu Wang, Yuanyuan Jiang, Wenzhong Wu

**Affiliations:** 1 Department of Acupuncture, Nanjing Hospital of Chinese Medicine Affiliated to Nanjing University of Chinese Medicine, Nanjing, Jiangsu, China; 2 Nanjing University of Chinese Medicine, Nanjing, Jiangsu, China; 3 Department of Acupuncture Rehabilitation, Affiliated Hospital of Nanjing University of Chinese Medicine, Nanjing, Jiangsu, China; The Second Affiliated Hospital of Shandong First Medical University, CHINA

## Abstract

**Objective:**

This systematic review aimed to clarify if acupuncture is more effective for menopausal insomnia compared with sham acupuncture, standard care (sedative hypnotics and/or MHT) or waitlist control.

**Methods:**

Seven literature databases were searched on April 30, 2024, to identify RCTs assessing the effectiveness of acupuncture. The methodological quality was assessed by the Cochrane Collaboration, and meta-analyses were conducted to calculate comparative effects using Rev Man software.

**Results:**

28 RCTs were analyzed. Six sham acupuncture-controlled RCTs were notable because of their high quality, and they showed that acupuncture significantly lowered PSQI scores, increased TST, sleep efficiency, and reduced WASO. The effect of acupuncture was maintained at a 4-week follow-up. Sixteen RCTs compared acupuncture with standard care, which showed acupuncture significantly reduced PSQI scores, KI scores, HAMD and HAMA scores. However, the subgroup analysis showed that there was no obviously difference between acupuncture and western medication in the treatment duration >8 weeks. Five RCTs assessed acupuncture combined with standard care and showed a favorable reduction in the PSQI score than standard care. One RCT showed that acupuncture significantly reduced PSQI and KI scores than a waitlist control. The GRADE assessment demonstrated that the level of evidence was very low to moderate, probably for the poor methodological quality and substantial heterogeneity among studies.

**Conclusion:**

The results showed that acupuncture may play a positive role in patients with menopausal insomnia.

## 1. Introduction

Aging and the transition through menopause are associated with increased prevalence of sleep disturbances in women. Menopausal insomnia is characterized as difficulty in falling asleep and/or a high frequency of waking at night [1], with a prevalence of 38%-50% during menopause [2,3]. The prevalence of insomnia was reported to increase from 33%-36% in premenopausal women to 61% in menopausal women [4,5]. In addition, insomnia is often more severe and longer lasting in menopausal women than in premenopausal women [6]. Long-term insomnia frequently causes daytime dysfunction, inattention, and emotional changes [7]. Moreover, it is associated with greater prevalence of anxiety and depression, aortic disease, coronary disease, and cardiovascular disease [8,9].

Because menopausal symptoms result from decreased estrogen levels, menopausal hormone therapy (MHT) is often recommended for the treatment of menopausal insomnia [10]. However, MHT carries some risks and side effects including breast cancer and cardiovascular events [11]. Therefore, some women are reluctant to use MHT. Benzodiazepine drugs are also effective and widely used for insomnia, but their adverse effects (AEs), especially dependence and increased risk of falls [12], may restrict their clinical application. Furthermore, a large prospective study revealed that 70% of patients using a prescription sleep aid continued its use at one year follow-up but did not demonstrate significant improvements in sleep compared with non-users [13]. Although cognitive behavioral therapy (CBT) is the first-line treatment for chronic insomnia, many people find it difficult to access because trained therapists are needed to address sleep disorders. Complementary and alternative medicine (CAM) is used by 22% to 61% of Western menopausal women [14]. Acupuncturists rank as the second most consulted practitioners by this population. Although more than three systematic reviews [14–16] have appraised the effects of acupuncture on insomnia in menopausal women, their conclusions were inconsistent. This is due to the wide variability in the interventions, quality, and outcomes analyzed in those studies. Some of the systematic reviews included randomized controlled trials (RCTs) with acupuncture, moxibustion, acupressure, or acupoint catgut embedding, which could contribute to greater heterogeneity of studies and make it difficult to reach reliable and accurate conclusions.

Therefore, we conducted a new systematic review focusing on the following topics: (1) Is acupuncture effective as an independent therapy for menopausal insomnia compared with a sham control or standard care for menopausal insomnia? (2) Is acupuncture effective as an adjuvant to standard care for menopausal insomnia? (3) What are the long-term effects of acupuncture compared with sham control or standard care?

## 2. Methods

### 2.1. Study registration

The systematic review and meta-analyses were conducted according to the Preferred Reporting Items for Systematic Reviews and Meta-Analyses. The study was registered on the PROSPERO database (https://www.crd.york.ac.uk/PROSPERO/) under the registration number: CRD42018092917.

### 2.2. Study eligibility

#### 2.2.1. Type of study

All clinical RCTs of acupuncture for managing menopausal insomnia were included, if published in Chinese or English. Case reports, clinical reviews, meeting abstracts, duplicate publications, animal experiments, and other trials lacking relevant outcome indicators were excluded.

#### 2.2.2. Participants

Menopausal stage was identified using the criteria of Stage of Reproductive Aging Workshop (STRAW) [17] or Obstetrics and Gynecology [18]. Diagnosis of insomnia was based on a version of Diagnostic and Statistical Manual of Mental Disorders, Fifth Edition (DSM-V) [19], Chinese Classification and Diagnostic Criteria of Mental Disorders, Third Edition (CCMD-3) [20], International Classification of Sleep Disorders, Third Edition (ICSD-3) [21], or International Classification of Sleep Disorders, Second Edition (ICSD-2) [22]. Participants need to meet the diagnosis of menopause and insomnia. Patients must be between 40 and 60 years old, regardless of their nationality, disease stage, or disease severity.

#### 2.2.3. Interventions

Interventions were restricted to traditional needle acupuncture (TNA) including manual acupuncture (MA) and electroacupuncture (EA) or TNA used alone or in combination with standard care (sedative hypnotics, MHT, and/or CBT) for menopausal insomnia. Control groups could receive Western medication (sedative hypnotics and/or MHT), CBT, sham acupuncture, or waitlist control.

#### 2.2.4. Outcomes

The following outcomes were assessed: (a) the primary outcome was the Pittsburgh Sleep Quality Index (PSQI); (b) perimenopausal-related symptoms were assessed using the Kupperman index (KI) or Menopause-Specific Quality of Life (MENQOL); (c) objective outcomes (polysomnography [PSG] or actigraphy), sleep-onset latency (SOL; in minutes), total sleep time (TST; in minutes), waking after sleep onset (WASO; total recording time-SOL-TST, in minutes), and percent sleep efficiency (SE; TST/total recording time × 100%); (d) depression/anxiety symptoms, and (e) safety, evaluated as the ratio of the number of reported AEs to the total number.

### 2.3. Search strategy

We performed systematic literature searches of PubMed, The Cochrane Library, Web of Science, EMBASE, China National Knowledge Infrastructure (CNKI), Wan Fang Database, China Biology Medicine (CBM), and the VIP Database from the date of database inception to April 30, 2024, to retrieve published RCTs describing the application of acupuncture for treatment of menopausal insomnia. The search consisted of a combination of MeSH terms and free words. The search strategy for PubMed and CNKI were provided in the S2 File.

### 2.4. Article screening and data extraction

Two researchers (XZ and CL) independently selected the studies and collected the data, importing the identified studies into EndNote X9.0. Where disagreements occurred, a third researcher (SQ) was consulted to reach a consensus. The following information was retrieved: Publication information (the last name of the first author, publication year, grouping methods, and number of women per group); the study population (age, duration of menopausal insomnia, diagnostic criteria, and TCM syndrome type of patients); details of the intervention (timing, frequency, and dose of acupuncture and the acupoints selected) and control treatment (timing, frequency, and dose of placebo/sham acupuncture or type, dose, and oral frequency of Western medications); outcome measures; and other data (follow-up duration and AEs). Funnel plots were used to evaluate publication bias for analyses where there were more than 10 eligible studies.

### 2.5 Risk of bias assessment

Two trained researchers (QW and YJ) independently assessed the risk of bias in the included studies using the Cochrane Collaboration’s risk of bias tool. If there were any disagreements, a third reviewer (WW) helped reach consensus. The evaluations included the following categories: Random sequence generation, allocation concealment, blinding of participants and personnel, blinding of outcome assessors, completeness of outcome data, selective reporting, and other bias. The risk of bias was rated as low, high, or unclear for each category.

### 2.6. Data analyses

The meta-analysis was performed using RevMan version 5.4 (Cochrane Collaboration). For continuous variables, such as the scores for sleep/perimenopause scales, we determined the mean difference (MD) with 95% confidence intervals (95% CI). For dichotomous variables (effective rate), we determined the relative risk (RR) with 95% confidence intervals (95% CI). The *I*^*2*^ statistic was used to assess the heterogeneity among RCTs. *I*^*2*^ > 50% indicates heterogeneity, and *I*^*2*^ < 50% was assumed to indicate no heterogeneity. In analyses where *P* > 0.1 and *I*^*2*^ < 50%, a fixed effects model was applied, otherwise a random effects model was used. If substantial heterogeneity was detected, subgroup and sensitivity analyses were considered to explore the causes of heterogeneity. If the sources of heterogeneity could not be determined, descriptive analyses were performed.

### 2.7 Evidence certainty assessment

To assess the evidence certainty, we used the GRADEpro online tool (http://gdt.gradepro.org/app#projects) to perform the evaluation and followed the recommended procedures for grading (high, moderate, low, very low).

## 3. Results

**Fig 1:**
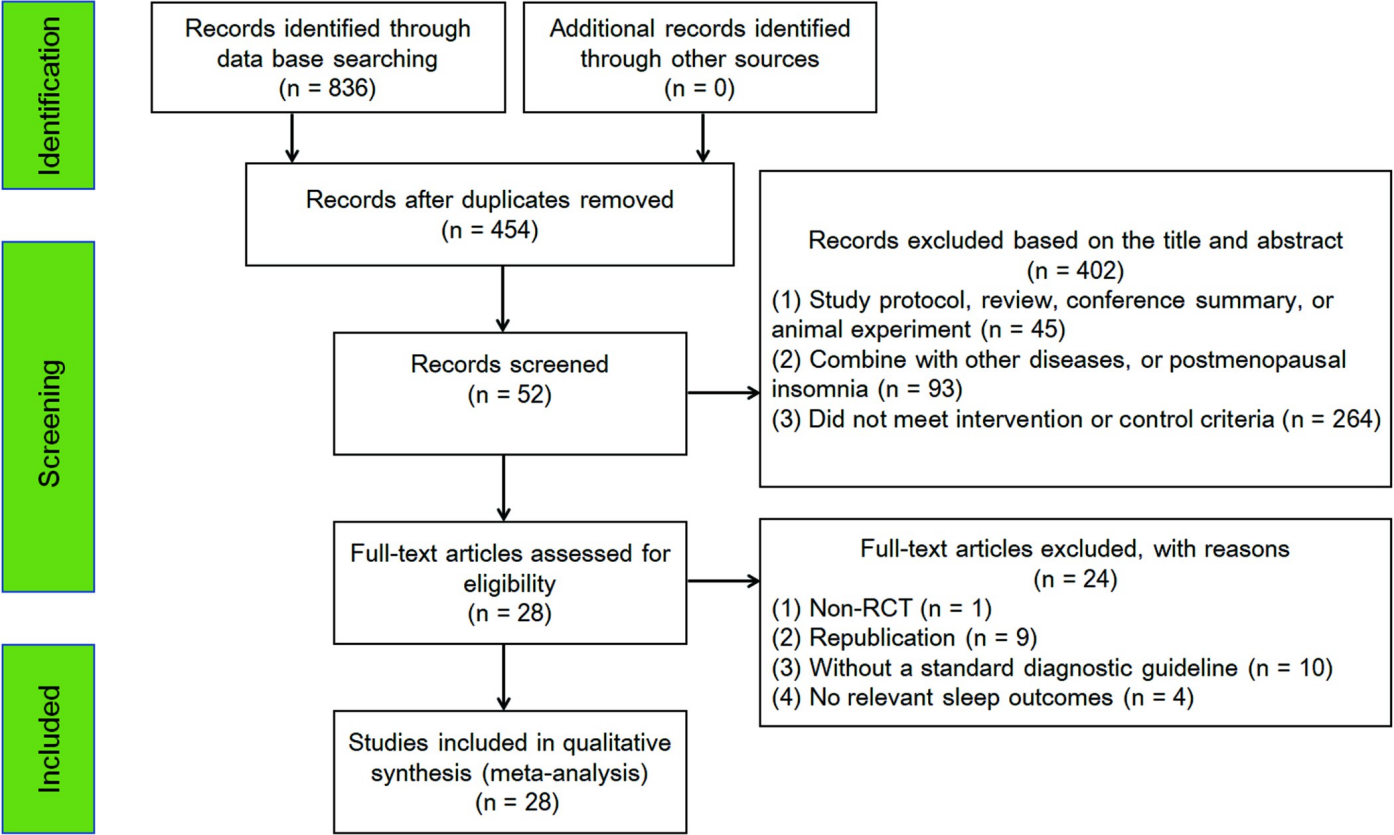
Study flow diagram. CBT, cognitive behavioral therapy; EA, electroacupuncture; MA, manual acupuncture; RCT, randomized controlled trial.

A total of 836 articles were initially retrieved by the searches. After removing the duplicates and further screening, 28 RCTs (involving 2,063 participants) were ultimately included in the meta-analyses. The literature screening process is summarized in Fig 1.

### 3.1. Study characteristics

The features of the included studies were shown in Table 1. All 28 RCTs [23–50] were conducted in China, of which 3 were published in English [23,24,28] and 25 in Chinese [25–27,29–50]. Four RCTs [24,26,33,41] investigated the clinical efficacy of EA, and the other 24 RCTs investigated the effects of MA. Six RCTs [23–28] compared acupuncture vs. sham acupuncture, 16 RCTs [29–44] compared acupuncture vs. standard care (sedative hypnotics and/or MHT), 5 RCTs [45–49] compared acupuncture as an adjuvant to standard care (sedative hypnotics and/or MHT) vs. standard care, and 1 RCT [50] compared acupuncture vs. a waitlist control group.

**Table 1:** Characteristics of the 28 studies included in the analyses.

Study(first name, year)	Cases (T/C)/n	Age (T/C)/year	insomnia duration (months = m, year s = y)	Diagnostic system	TCM syndrome type	Treatment group		Control group	Outcome measure	Follow-up times	Adverse events (T/C)/n
						Type	Mian acupuncture points				
Fu 2017 [23]	T/n = 37 C/n = 37	T/ 52.0 ± 5.3 C/52.5 ± 5.9	NR	-STRAW -ICSD-3	NR	MA; -3 times/week for 3 weeks	BL23, BL18, LR14, GB25;	Sham-acupuncture;	PSQI, PSG, ISI	The 30th day	T/n = 0; C/n = 4 [worsening of insomnia (4)];
Li 2020 [24]	T/n = 40 C/n = 41	T/52.12 ± 4.19 C/53.0 ± 3.81	T/39.19 m C/40.21 m	-STRAW -ICSD-3	Kidney yin deficiency and Kidney yang deficiency	EA; -18 times over the course of 8 weeks (3 times per week for 4 weeks, twice per week for 2 weeks, once per week for 2 weeks) -continuous wave type, frequency at 2.5 HZ	GV20, GV24, GV29, RN6, RN4, EX-HN22, SP6, HT7	Sham- acupuncture;	PSQI, Men-QoL, actigraphy, ISI	The 4th week, and the 12th week	T/n = 2 [bleeding (1), pain (1)]; C/n = 1 [pain (1)];
Yang W.J. 2017 [25]	T/n = 37 C/n = 36	T/54.5 C/54.5	T/19.47 ± 7.65 m C/17.5 ± 9.04 m	-STRAW -ICSD-2 -CDTE-TCM	Deficiency of kidney yin	MA; -3 times/week for 10 times	BL23, EX-HN22, KI3, LR3	Sham-acupuncture;	PSQI, ISI, BDI, BAI, PSG	The 30th day	NR
Yu 2022 [26]	T/n = 30 C/n = 30	T/49.7 ± 3.2 C/48.8 ± 3.4	T/12.2 ± 4.7 m C/11.6 ± 4.5 m	-Obstetrics and Gynecology -ICSD-3 -CDTE-TCM	Yin deficiency of liver and kidney	EA; -3 times/week for 4 weeks -continuous wave type, frequency at 2.5 HZ	EX-HN5, EX-HN3, ST25, CV4, BL18, BL23, KI3, LR3	Sham-acupuncture;	PSQI, KI	NO follow-up	NR
Yang W.J. 2023 [27]	T/n = 35 C/n = 35	T/53 ± 4 C/52 ± 4	T/11.37 ± 3.60 m C/10.77 ± 3.21 m	-STRAW -ICSD-3 -CDTE-TCM	Deficiency of kidney yin	MA; -3 times/week for 10 times	BL23, EX-HN22, KI3, GV20	Sham-acupuncture;	PSQI, PSG	NO follow-up	NR
Zhao F.Y. 2023 [28]	T/n = 35 C/n = 35	T/48.94 ± 2.25 C/48.80 ± 2.07	T/13.37 ± 2.20 m C/12.77 ± 2.90 m	-STRAW -ICSD-3 -CDTE-TCM	Liver depression and kidney deficiency	MA; -8 weeks for 17 times (3 times per week for 3 weeks, twice per week for 3 weeks, once per week for 2 weeks	EX-HN3, GV20, CV4, CV7, PC6, KI3, LR3, SP6, EX-CA1	Sham-acupuncture;	PSQI, HAMD, HAMA, Men-QoL	The 8th week and the 16th week	T/n = 6 [bleeding (2), pain (4)]; C/n = 3 [pain (1), dizziness (2)];
Yan 2021 [29]	T/n = 42 C/n = 43	T/51.5 ± 4.6 C/50.9 ± 4.2	T/8.9 ± 5.6 m C/9.1 ± 6.1 m	-Obstetrics and Gynecology -CCMD-3	NR	MA; -7 times/week for 12 weeks	GV20, GV24, CV6, CV4, HT7, SP6	Estazolam; -1 mg/day for 12 weeks	PSQI, MENQOL	NO follow-up	T/n = 3 [bleeding (1), dizziness (2)]; C/n = 2 [dizziness (1), daytime sleeping (1)];
Luo 2020 [30]	T/n = 30 C/n = 30	All participants: 46.8 ± 7.3	All participants: 3–20 m	-Obstetrics and Gynecology -CCMD-3 -CDTE-TCM	Heart-kidney disharmony	MA; -5 times/week for 12 weeks	GV20, EX-HN1, BL62, KI6, EX-HN22, PC6, SP6, K I3, BL15, BL23	Estazolam; -1 mg/day for 12 weeks	PSQI	NO follow-up	T: A few cases felt pain and bleeding; C/n = 5 [dizziness (2), trointestinal reaction (2), rash (1)];
Zhang 2017 [31]	T/n = 31 C/n = 30	T/50.45 ± 3.50 C/48. 97 ± 2. 88	T/20.38 ± 20.53 m C/20.36 ± 20. 44 m	-Obstetrics and Gynecology -CCMD-3 -CDTE-TCM	Six syndromes with liver	MA; -5 times/week for 4 weeks	EX-HN22, BL17, BL18, LR3, GV20, EX-HN1	Estazolam; -1 mg/day for 4 weeks	PSQI, KI, HAMD, HAMA	3 months follow- up	T/n = 1 [hematoma (1); C/n = 4 [dizziness, fatigue, and daytime sleeping (2), memory loss (2)];
Qin 2018 [32]	T/n = 34 C/n = 33	T/51.97 ± 2.27 C/50.85 ± 2.77	T/18.44 ± 7.55 m C/20.58 ± 9.25 m	Obstetrics and Gynecology—CCMD-3 -CDTE-TCM	Hyperactivity of Liver and deficiency of kidney	MA; -5 times/week for 4 weeks	BL18, BL17, LR3, EX-HN22, BL23, EX-HN1, KI3, GV20	Estazolam; -1 mg/day for 4 weeks	PSQI, HAMA, PSG	NO follow-up	T/n = 5 [bleeding (3), lethargy (2)]; C/n = 5 [dizziness (2), fatigue (3)];
Ma 2014 [33]	T/n = 45 C/n = 45	T/50.04 ± 2.67 C/50.42 ± 2.96	T/13.36 ± 7.47 m C/13.51 ± 7.76 m	Obstetrics and Gynecology -CCMD-3 CDTE-TCM	Fire transformation of liver stagnation; Phlegm-heat attacking internally; Yin deficiency fire; Heart-Spleen Deficiency	EA; -3 times/week for 4 weeks -Continuous dense wave, > 50HZ	PC6, SP6, Si shen zhen (1.5 cun apart from GV20), Ding shen zhen (0.5 cun up to EX-HN3, and 0.5cun up to GB14)	Estazolam; -1 mg/day for 4 weeks	PSQI, HAMD	NO follow-up	NR
Kang 2015 [34]	T/n = 31 C/n = 33	T/47.5 ± 4.2 C/49.2 ± 3.9	T/15.9 ± 6.7 m C/16.6 ± 6.3 m	-Obstetrics and Gynecology -CCMD-3 -CDTE-TCM	Qi deficiencies of the heart and gall	MA; -6 times/week for 4 weeks	GV20, EX-HN1, EX-HN22, GB20, GB13, GV24	Estazolam; -2 mg/day for 4 weeks	PSQI, KI	NO follow-up	T/n = 0; C/n = 1 [nausea (1)]
Yang J.R. 2017 [35]	T/n = 81 C/n = 81	T/48.17 ± 4.12 C/49.45 ± 3.98	T/7.13 ± 1.96 m C/7.53 ± 2.11 m	-Obstetrics and Gynecology -CCMD-3 CDTE-TCM	Yin deficienc-y of liver and kidney	MA; -every other day,15 times as a course, for 3 courses	PC6, HT7, ST36, KI3, ST40, CV12	Estazolam; -1 mg/day for 3 menstrual cycles	PSQI	NO follow-up	NR
Lai 2016 [36]	T/n = 34 C/n = 33	T/51.28 ± 4.19 C/51.47 ± 4.03	T/8.33 ± 3.85 m C/9.08 ± 3.83 m	-Obstetrics and Gynecology -CCMD-3 -CDTE-TCM	Heart-kidney disharmony	MA; -6 times/week for 3 weeks	BL62, SI3, KI6, LU7	Zopiclone; -3 mg/day for 3 weeks	PSQI, KI	NO	T/n = 0; C/n = 3 [adverse drug reactions (3)];
Li 2018 [37]	T/n = 60 C/n = 62	T/51 ± 4 C/50 ± 4	T/11.2 ± 5.2 m C/10.2 ± 5.3 m	-Obstetrics and Gynecology -CCMD-3	NR	MA; -5 times/week for 9 weeks	BL13, BL15, BL16, BL17, BL20, BL23, HT7	Alprazolam; -0.4–0.8 mg/day for 9 weeks	PSQI	NO follow-up	NR
Guo 2021 [38]	T/n = 30 C/n = 30	T/49.38 ± 3.65 C/50.20 ± 4.1	T/36.53 ± 13.61 m C/33.93 ± 13.69 m	-Obstetrics and Gynecology -DSM-5	NR	MA; -6 times/week for 8 weeks	EX-HN3, CV6, CV4, CV12, GV20, GV24, SP6, ST36, PC6, HT7	Estazolam; -1 mg/day for 8 weeks	PSQI, KI	NO follow-up	NR
Dai 2023 [39]	T/n = 18 C/n = 20	T/52.21 ± 4.18 C/52.49 ± 4.05	NR	-Obstetrics and Gynecology -ICSD-3	Yin deficiency of kidney	MA; -6 times/week for 4 weeks	GV20, BL23, KI6, BL62, EX-HN22, KI3	Zopiclone; -7.5 mg/day for 4 weeks	PSQI	NO follow-up	NR
Liao 2023 [40]	T/n = 30 C/n = 30	T/49.83 ± 3.11 C/49.10 ± 2.81	NR	-Obstetrics and Gynecology -ICSD-3 -CDTE-TCM	Incoordination between heart and kidney	MA; -5 times/week for 2 weeks	EX-HN3, GB14, CV4, ST36, SP6, CV6, PC6, HT7, PC8, KI1	Estazolam; -1 mg/day for 2 weeks	PSQI	NO follow-up	NR
Liu 2023 [41]	T/n = 28 C/n = 27	T/50.33 ± 2.55 C/50.63 ± 3.01	T/16.80 ± 11.86 m C/16.53 ± 11.82 m	-Obstetrics and Gynecology -CCMD-3 -CDTE-TCM	Kidney deficiency and liver depression	EA; -5 times/week for 4 weeks—dense wave, > 0.7HZ	EX-HN3, BL15, BL23, CV4, CV6, ST25, ST29, KI3, LI4, LR3	Alprazolam; -0.4 mg/day for 4 weeks	PSQI, KI	The 30th day	NR
ZhaoM 2023 [42]	T/n = 40 C/n = 40	T/49.85 ± 2.46 C/50.17 ± 2.71	T/5.13 ± 2.03 m C/6.04 ± 2.24 m	-Obstetrics and Gynecology -DSM-5	NR	MA; -5 times/week for 4 weeks	GV20, EX-HN1, EX-HN22, SP6, CV4, HT7, KI6, LR3	Estazolam; -1 mg/day for 4 weeks	PSQI	NO follow-up	NR
Tong 2023 [43]	T/n = 30 C/n = 30	T/47.38 ± 6.11 C/48.25 ± 6.32	T/21.45 ± 5.10 m C/21.26 ± 5.28 m	-Obstetrics and Gynecology -CCMD-3 -CDTE-TCM	Heart-kidney disharmony	MA; -3 times/week for 3 weeks	EX-HN1, PC6, SP6, Ding shen zhen (0.5 cun up to EX-HN3, and 0.5 cun up to GB14)	Estazolam; -1 mg/day for 3 weeks	PSQI, HAMA, HAMD	NO follow-up	T/n = 1 [dizziness (1)]; C/n = 4 [dry mouth (1), dizziness (1), nausea (1), diarrhea(1)];
Zhang2024 [44]	T/n = 36 C/n = 36	T53.4 ± 4.8 m C/52.1 ± 4.5 m	T/37.4 ± 2.85 m C/40.6 ± 15.3 m	-Obstetrics and Gynecology—DSM-5	NR	MA; -3 times/week for 4 weeks	GV20, LR3, KI3, KI12, HT5, ST36, CV3, EX-CA1	HRT; -0.312mg/day for 4 weeks	PSQI	NO follow-up	NR
Zhu 2016 [45]	T/n = 37 C/n = 37	T/49.86 ± 3.15 C/49.27 ± 3.58	T/2.99 ± 4.24 y C/2.97 ± 3.42 y	-Obstetrics and Gynecology -CCMD-3	NR	MA + Estazolam; -5 times/week for 4 weeks	CV12, EX-HN1, GV20, GV24, HT7, KI3, LR3, SP9, ST25;	Estazolam; -1 mg/day, 5 days/week for 4 weeks	PSQI	NO follow-up	NR
Gao 2014 [46]	T/n = 32 C/n = 32	T/49.13±2.47 C/49.50 ± 2.51	T/6.00 ± 3.12 m C/5.88 ± 2.70 m	-Obstetrics and Gynecology -CCMD-3	NR	MA + Estazolam; -6 times/week for 4 weeks	Ex-B2	Estazolam; -2 mg/day for 4 weeks	PSQI	NO follow-up	NR
Ma 2016 [47]	T/n = 35 C/n = 35	T/49.80 ± 3.22 C/50.34 ± 2.99	T/10.74 ± 6.95 m C/10.91 ± 7.197 m	-Obstetrics and Gynecology -CCMD-3	NR	MA + Estazolam; -1 time/day for 4 weeks	KI7, LR3, KI3, KI10, SP6, HT7, EX-HN22, ST36, SP10	Estazolam; -2 mg/day for 4 weeks	PSQI	NO follow-up	NR
Han 2023 [48]	T/n = 38 C/n = 38	T/51.77 ± 2.37 C/52.02 ± 2.45	T/18.02 ± 3.35 m C/17.93 ± 3.18 m	-Obstetrics and Gynecology -CCMD-3 -CDTE-TCM	NR	MA + Estazolam; -1 time/day for 8 weeks	CV6, CV4, CV12, CV10, ST25, GV24, EX-HN3, EX-HN1, GB13, SP6, HT7, LR3, LR14, KI6, BL62	Estazolam; -1 mg/day for 8 weeks	PSQI, PSG, KI, HAMA, HAMD	NO follow-up	NR
Xue 2023 [49]	T/n = 42 C/n = 41	T/48.35 ± 2.37 C/47.75 ± 3.10	T/7. 34 ± 1. 63 m C/8.02 ± 1.46 m	-Obstetrics and Gynecology -DSM-5	Liver spleen deficiency	MA + Estazolam; -3 times/week for 4 weeks	EX-HN1, EX-HN22, GV20, BL62, LI4, ST40, LR2, LR3, LR14, KI6, SP6, BL18	Estazolam; -1 mg/day for 4 weeks	PSQI, KI, HAMA	NO follow-up	T/n = 5 [daytime sleeping (2), dizziness (1), fatigue (1), dry mouth (1)]; C/n = 10 [daytime sleeping (4), dizziness(3), fatigue(2), dry mouth (1)];
Lin 2017 [50]	T/n = 33 C/n = 32	T/50 ± 3 C/50 ± 3	T/14.16 ± 13.08 m C/14.63 ± 10.83 m	-Obstetrics and Gynecology -CCMD-3	NR	MA; -3 times/week for 4 weeks	CV6, CV4, RN12, GV20, GV24, GV29, SP6, ST36, PC6, HT7	Waitlist control;	PSQI, KI	NO follow-up	NR

AIS, Athens Insomnia Scale; BAI, Beck Anxiety Inventory; BDI, Beck Depression Inventory; C, control; CCMD-3, Chinese Classification of Mental Disorders version 3; CDTE-TCM, Criteria of Diagnosis and Therapeutic Effect of Diseases and Syndromes in TCM; DSM-5, Diagnostic and statistical manual of mental disorders version 5; EA, electroacupuncture; HAMA, Hamilton Rating Scale for Anxiety; HAMD, Hamilton Rating Scale for Depression; HRT, hormone replacement therapy; ICSD-2, International Classification of Sleep Disorders version 2; ICSD-3, International Classification of Sleep Disorders version 3; KI, Kupperman index; MA, manual acupuncture; MENQOL, Menopause-Specific Quality of Life; MRS, Menopause Rating Scale; NR, not reported; PSQI, Pittsburgh Sleep Quality Index; PSG, polysomnography; QoL, quality of life; T, treatment; TCM, traditional Chinese medicine.

The frequency of treatment ranged from 1 to 7 sessions per week, with the most common being 3 sessions per week (11/28 studies). Although the duration of treatment varied considerably, ranging from 21 to 90 days, 85.7% of the studies (24/28) involved a treatment duration of ≥4 weeks. In all 28 studies, a total of 56 acupoints were used as the main acupoints. The 10 most frequently used acupoints were GV20 (56.5%), HT7 (52.2%), SP6 (39.1%), BL23 (34.5%), KI3 (34.8%), EX-HN22 (34.8%), LR3 (30.4%), PC6 (30.4%), CV4 (30.4%), and GV24 (26.1%).

### 3.2. Risk of bias assessment

**Fig 2:**
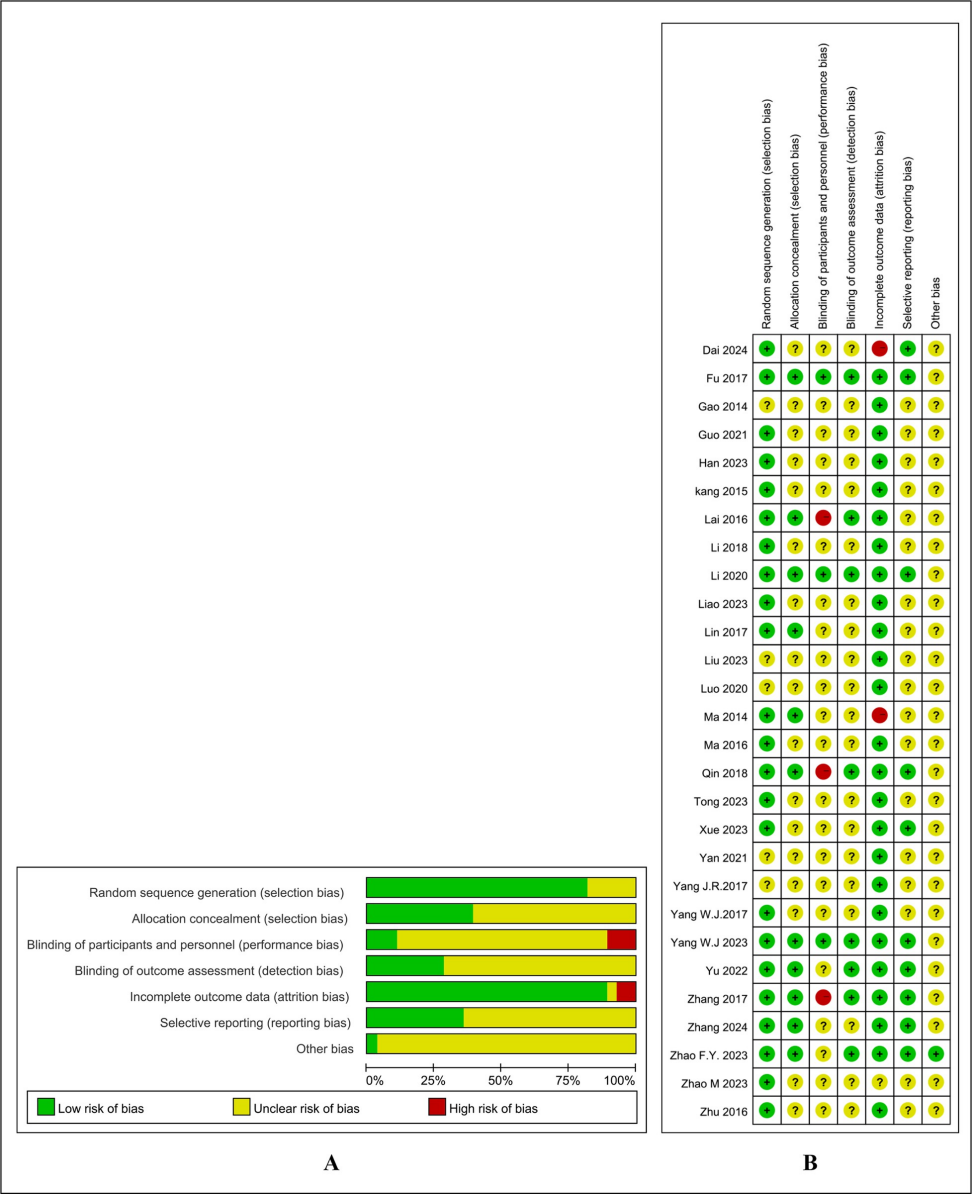
A: Risk of bias overall. B: Risk of bias in individual studies.

Parameters of quality assessment included the following. (a) Random sequence generation: 23 RCTs [23–28,31–34,36–45,47,49,50] clearly described the methods of randomization. The other seven RCTs did not specify the methods of randomization. (b) Allocation concealment: 12 RCTs [23–28,31–33,36,44,50] were judged to have a low risk of bias for this item, but this could not be determined in the other 17 studies [29,30,34,35,37–43,45–49]. (c) Blinding of participants and personnel: Because acupuncture must be performed by a qualified professional, it was impossible to blind the acupuncturists. Only six of the RCTs [23–28] using sham acupuncture as a control involved blinding of the patients. (d) Blinding of outcome assessment: Nine RCTs [23–28,31,32,36] involved blinding of the data evaluators. (e) Incomplete outcome data: Two RCT [33,39] were rated to show high risk of bias and the rest as low risk of bias for incomplete outcome data. (f) Selective reporting: Nine RCTs [23–28,39,41,44] showed a low risk of bias because their protocols were registered on the Chinese Clinical Trial Registry (ChiCTR); the other RCTs were rated as having unclear risk. (g) None of the RCTs described other sources of bias and were rated as showing unclear risk (Fig 2).

### 3.3. Outcomes of acupuncture

The 28 RCTs could be divided into four groups based on the comparator used: (1) Acupuncture vs. Sham acupuncture (6 RCTs); (2) Acupuncture vs. Western medication (sedative hypnotics and/or MHT; 16 RCTs); (3) Acupuncture plus Western medication vs. Western medication (5 RCTs); and (4) Acupuncture vs. Waitlist control (1 RCT).

#### 3.3.1. Acupuncture vs. sham acupuncture

Six RCTs [23–28] (*n* = 428) were included in this comparison. We performed meta-analyses for the outcome measures PSQI, polysomnography, and actigraphy. Other outcome measures could not be analyzed because there were fewer than three RCTs per outcome.

**Fig 3:**
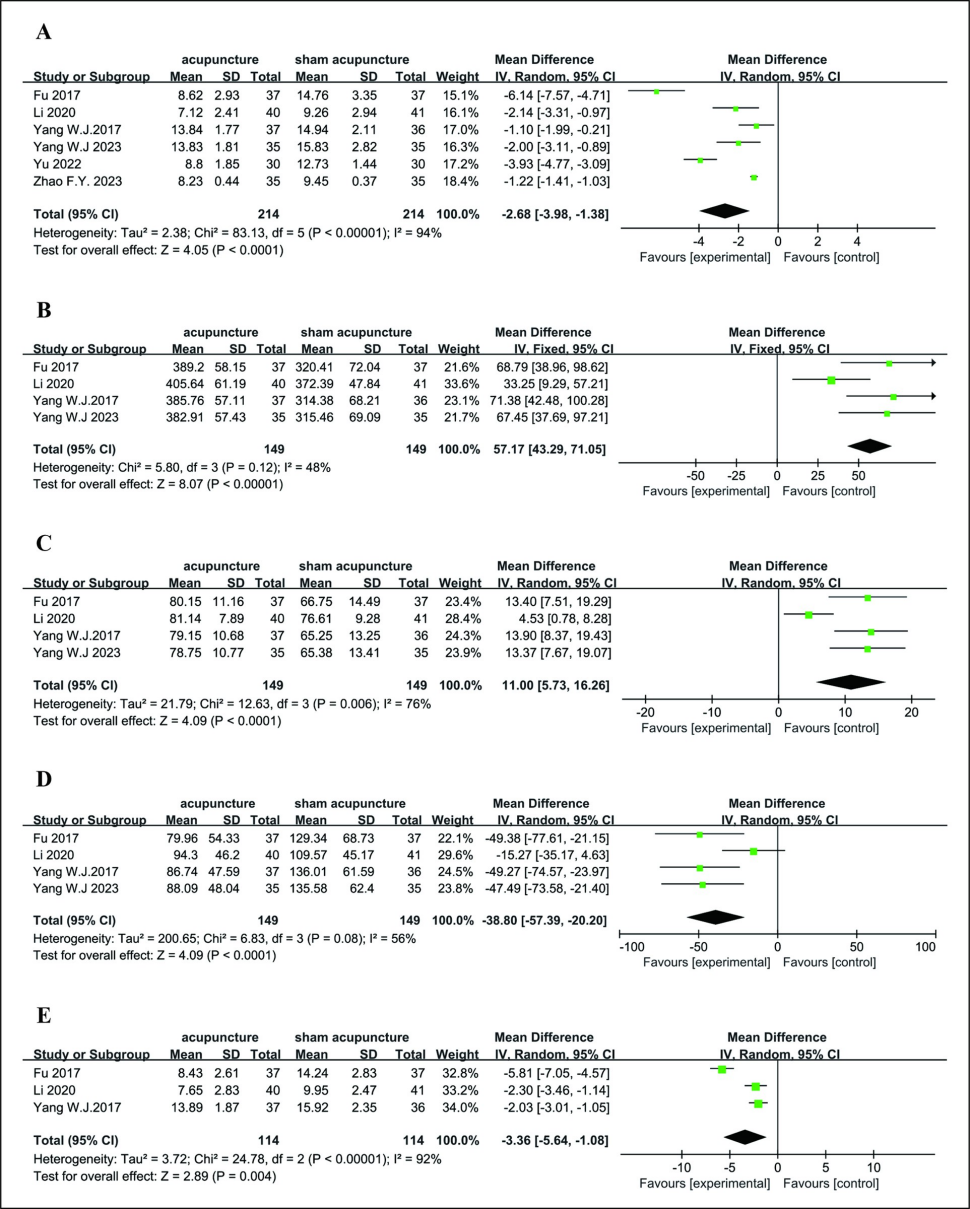
A: Forest plots of acupuncture vs. sham acupuncture for PSQI after treatment; B: Forest plots of acupuncture vs. sham acupuncture for TST after treatment; C: Forest plots of acupuncture vs. sham acupuncture for SE after treatment; D: Forest plots of acupuncture vs. sham acupuncture for WASO after treatment; E: Forest plots of acupuncture vs. sham acupuncture for PSQI at the 4-week follow-up.

*3*.*3*.*1*.*1*. *PSQI*. PSQI scores were reported in all six RCTs [23–28]. The results of the meta-analysis revealed that acupuncture was associated with significantly lower PSQI scores than sham acupuncture (MD = −2.68 [95% CI −3.98, −1.38], *P* < 0.0001, *I*^*2*^ = 94%). Because there was high heterogeneity, the random effect model was used (Fig 3A).

*3*.*3*.*1*.*2*. *Objective sleep parameters*. We analyzed TST, SE, and WASO as objective sleep parameters [23–25,27]. SOL could not be determined because it was not reported in an adequate number of RCTs. The meta-analyses demonstrated that the effects of acupuncture were superior to those of sham acupuncture for the outcomes of TST (MD = 57.17 [95% CI 43.29, 71.05], *P* < 0.0001, *I*^*2*^ = 48%), SE (MD = 11.0 [95% CI 5.73, 16.26], *P* < 0.00001, *I*^*2*^ = 76%), and WASO (MD = −38.80 [95% CI −57.39, −20.20], *P* < 0.0001, *I*^*2*^ = 56%) (Fig 3B–3D).

*3*.*3*.*1*.*3 Follow-up*. Three RCTs [23–25] compared the PSQI scores at a 4-week follow-up after acupuncture or sham acupuncture. The random effects model was used because of the heterogeneity of these RCTs (*P* < 0.00001, *I*^*2*^ = 92%). At the 4-week follow-up, acupuncture was associated with lower PSQI scores than sham acupuncture (MD = −3.36 [95% CI −5.64, −1.08], *P* = 0.004) (Fig 3E), whereas one RCT [28] reported acupuncture did not show any significant reduction of the PSQI compared with sham acupuncture at a 16-week follow-up.

*3*.*3*.*1*.*4 Subgroup analyses*. Subgroup analyses were performed for the high heterogeneities in all outcome measures. We selected the studies that presented the PSQI score, and divided these into subgroups based on 2 criteria: the acupuncture method, the total treatment duration. Regarding treatment duration (≤4 weeks or 8 weeks) (S1 Fig) or acupuncture methods (MA or EA) (S2 Fig), acupuncture was significantly more effective than sham acupuncture in both subgroups. But all the outcomes showed there was still high heterogeneity among the trials.

#### 3.3.2. Acupuncture vs. western medication

Sixteen RCTs compared acupuncture vs. western medication [29–44] (*n* = 1203). Meta-analyses were performed for PSQI, KI, HAMA and HAMD, but not for other outcomes reported in fewer than three RCTs.

**Fig 4:**
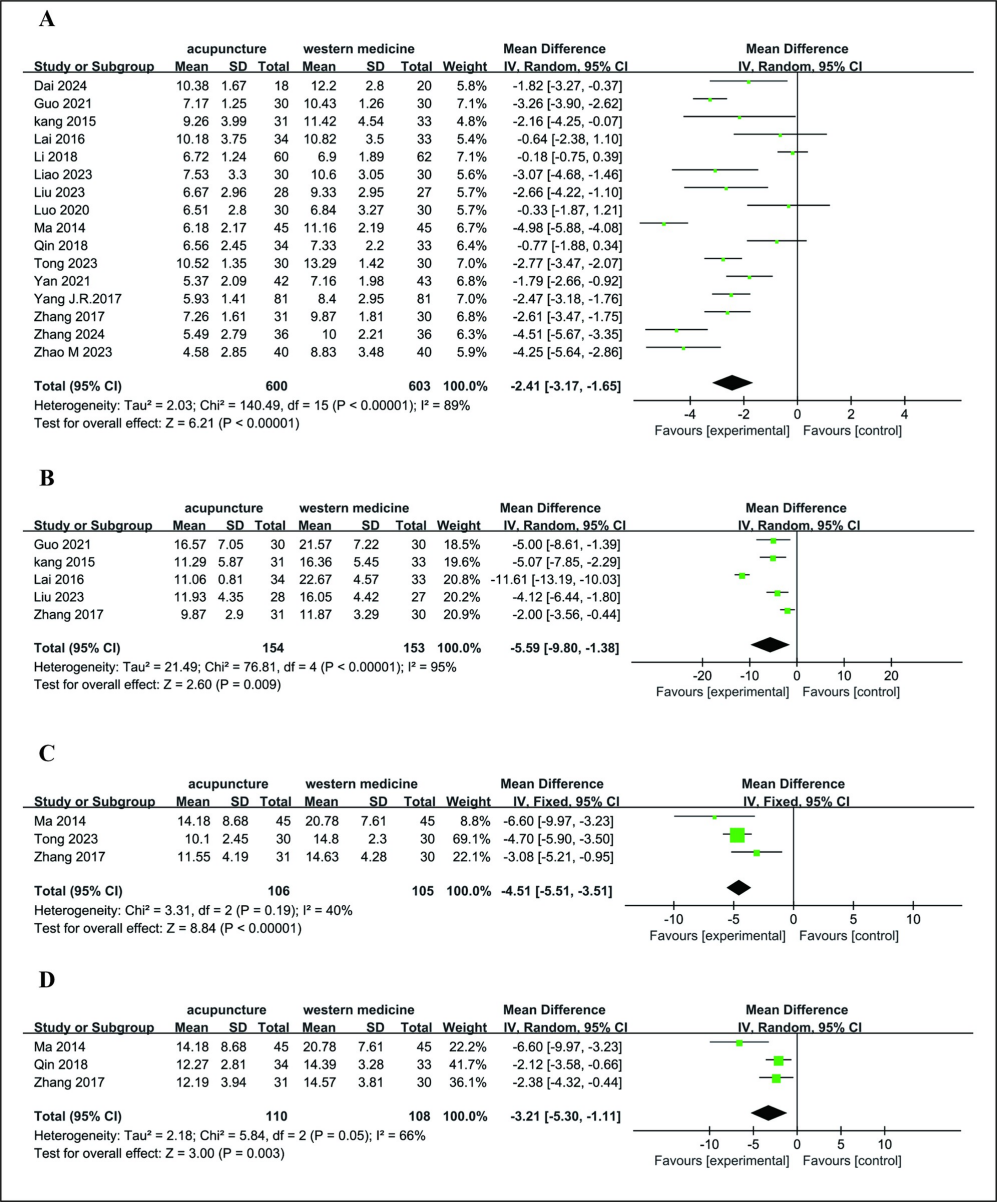
A: Forest plots of acupuncture vs. western medication for PSQI after treatment; B: Forest plots of acupuncture vs. western medication for KI after treatment; C: Forest plots of acupuncture vs. western medication for HAMD after treatment; D: Forest plots of acupuncture vs. western medication for HAMA after treatment.

*3*.*3*.*2*.*1*. *PSQI*, *KI*, *HAMA*, *and HAMD*. PSQI scores were reported in 16 RCTs [29–44]. In this analysis, the PSQI score was lower for acupuncture vs. western medication (MD = −2.41 [95% CI −3.17, −1.65], *P* < 0.00001, *I*^*2*^ = 89%) (Fig 4A). Five RCTs [31,34,36,38,41] reported changes in KI scores, and these showed better effects in decreasing KI scores (MD = −5.59 [95% CI −9.80, −1.38], *P* = 0.009, *I*^*2*^ = 95%) (Fig 4B). It also revealed that acupuncture could significantly improve the HAMD score [31,33,43] (MD = −4.51 [95% CI −5.51, −3.51], *P* <0.00001, *I*^*2*^ = 40%) (Fig 4C) and the HAMA score [31,32,43] (MD = −3.21 [95% CI −5.30, −1.11], *P* = 0.003, *I*^*2*^ = 66%) (Fig 4D).

*3*.*3*.*2*.*2*. *Subgroup analyses*. Subgroup analyses were also performed in the PSQI score. In terms of acupuncture method (MA or EA), acupuncture has better effects on the reduction of PSQI scores than western medication (S3 Fig). Regarding treatment duration, acupuncture showed significantly superior results compared with western medication in the treatment duration ≤4 weeks or 8 weeks. However, the subgroup analysis showed there was no obvious difference between acupuncture and western medication in the treatment duration >8 weeks [29,30,35,37] (MD = −1.24 [95% CI −2.54, 0.06], *P* = 0.06, *I*^*2*^ = 91%) (S4 Fig).

*3*.*3*.*2*.*3*. *Sensitivity analyses*. Sensitivity analyses were performed to identify the potential sources of heterogeneity and to examine the stability of the results. We only performed sensitivity analyses for the outcome PSQI scores because there were too few RCTs reporting data for the other outcome measures (all <10). In a leave-one-out analysis in which one RCT was excluded in each analysis, the overall combined results for the PSQI score did not change substantially (S5 Fig). This indicates that the results were robust, and no single study had a significant impact on the overall results.

#### 3.3.3. Acupuncture as an adjuvant to western medication vs. western medication

**Fig 5:**
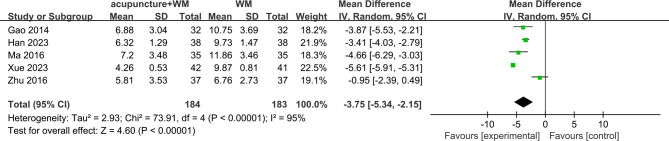
Forest plot of acupuncture as an adjuvant to western medication vs. western medication for PSQI. (WM, Western Medication).

Five RCTs [45–49] (*n* = 367) compared acupuncture as an adjuvant to western medication vs. western medication. We only performed a meta-analysis of PSQI scores because fewer than three RCTs reported each of the other outcomes. The results favored acupuncture as an adjuvant to western medication for PSQI scores (MD = −3.75 [95% CI −5.34, −2.15], *P* < 0.00001, *I*^*2*^ = 95%) (Fig 5). Subgroup analysis also showed that acupuncture combination western medication is statistically significantly better than single western medication after treatment duration ≤4 weeks or 8 weeks. But it still had comparably high levels of heterogeneity (S6 Fig).

#### 3.3.4. Acupuncture vs. waitlist control

One RCT [50] (*n* = 65) showed that acupuncture was associated with significant reductions in PSQI and KI scores compared with a waitlist control. The findings suggested that acupuncture can improve sleep quality and relieve menopausal symptoms in women with insomnia.

### 3.4. Publication bias

**Fig 6:**
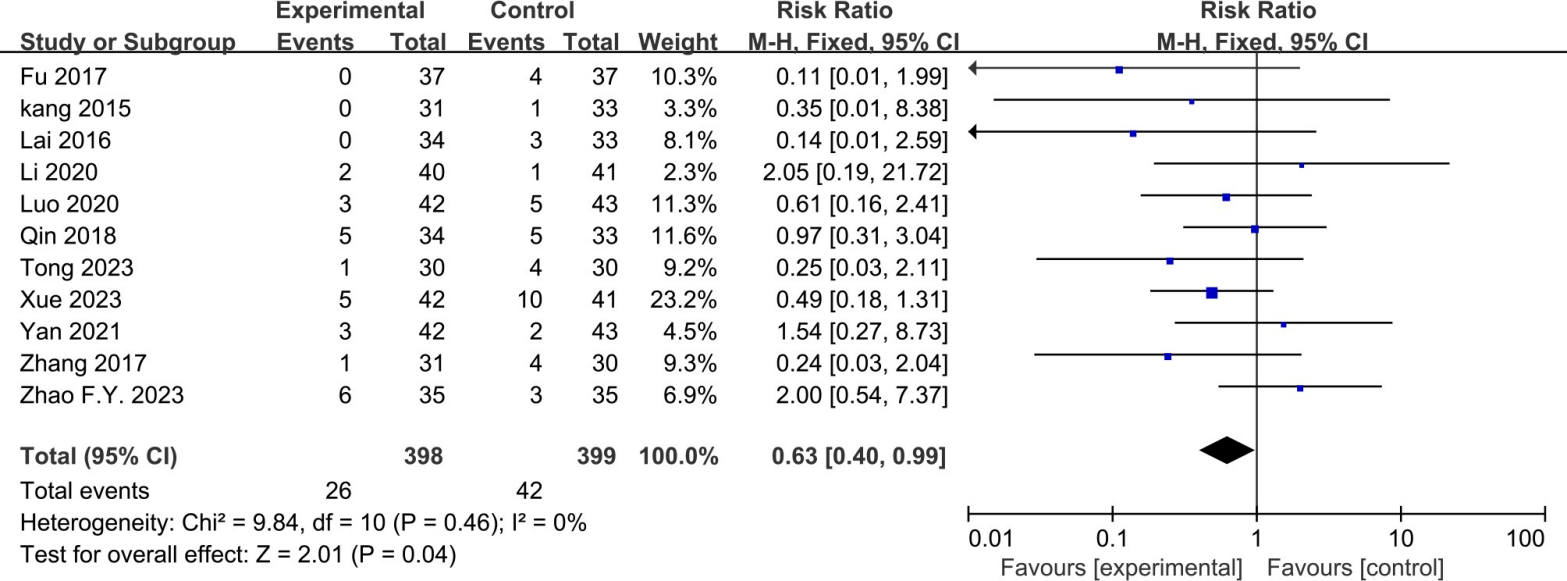
Forest plots of adverse event rates.

To assess publication bias, we prepared a funnel plot for one outcome, the PSQI score, because this outcome was reported in more than 10 RCTs. The funnel plot showed no apparent asymmetry (Fig 6). However, because all of the included studies were performed in China, we consider there is potential for publication bias as triggered by cultural background of different regions and countries. Accumulating evidence suggests that the Asia-Pacific region is more inclined towards acupuncture treatment and publishes positive results.

### 3.5. Safety

**Fig 7:**
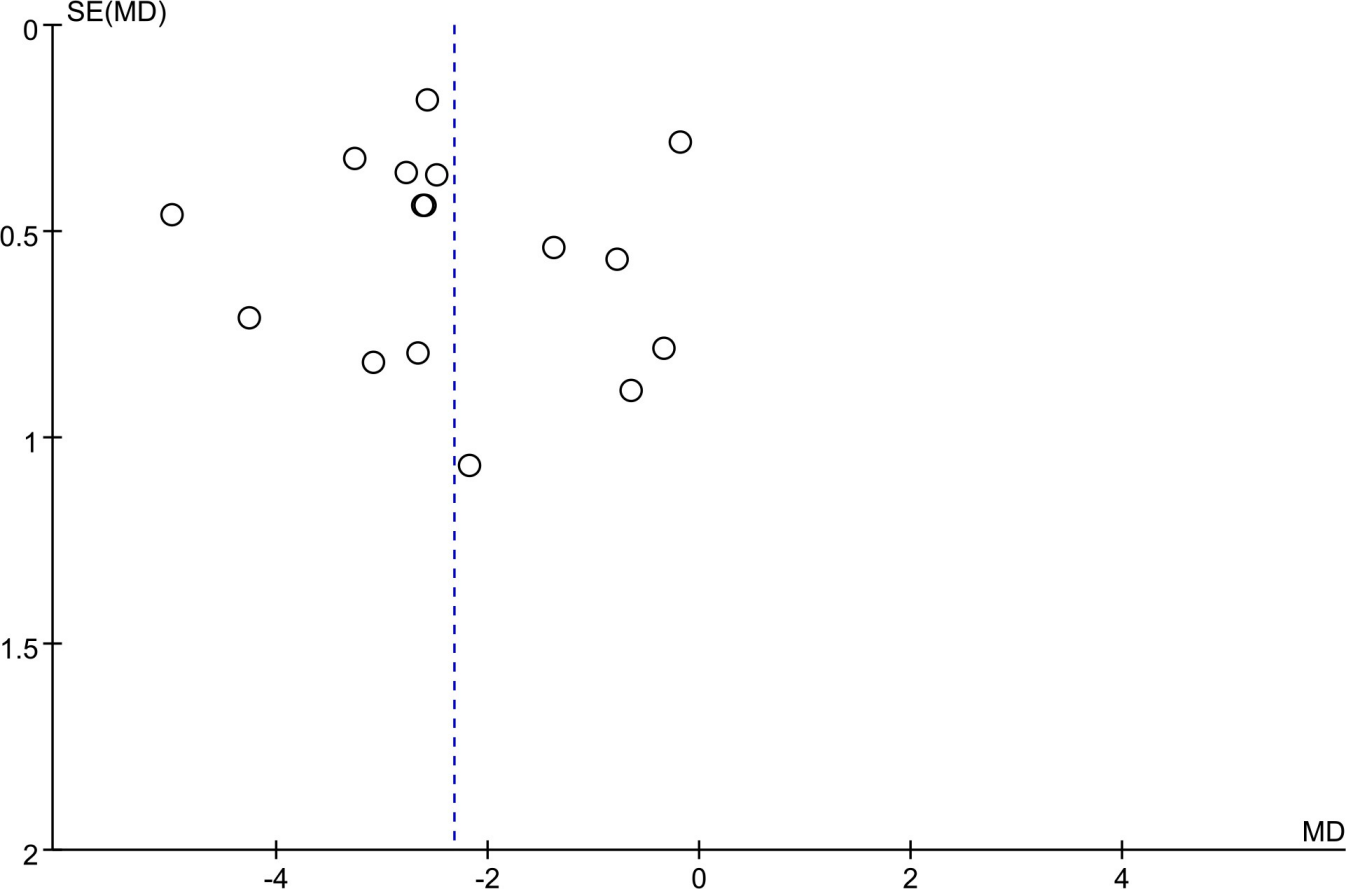
Funnel plot of publishing bias.

Eleven RCTs [23,24,28–32,34,36,43,49] provided information on AEs. The rate of AEs differed significantly between acupuncture and Western medication (RR = 0.63 [95% CI 0.40, 0.99], *P* = 0.04, *I*^*2*^ = 0%; Fig 7). The predominant adverse events were skin needle pain and subcutaneous tissue disorder at the acupuncture site. None of the RCTs reported any dropouts due to AEs associated with acupuncture.

### 3.6. Quality assessment

We evaluated the available evidence with the GRADE tool; the quality of evidence on acupuncture for menopausal insomnia was graded as “moderate, low or very low”. Details were shown in the S1 Table.

## 4. Discussion

### 4.1. Summary of main outcomes

In this systematic review and meta-analysis, acupuncture was associated with a greater reduction in PSQI scores, increase in TST, sleep efficiency, and a reduction in WASO vs. sham acupuncture. Acupuncture was also associated with better effects vs. sham acupuncture in terms of the decrease in the global PSQI score at a 4-week follow-up, suggesting that acupuncture may have long-term therapeutic effects on menopausal insomnia. It indicated that acupuncture appeared to possess marginally better efficacy than sham acupuncture. This conclusion is largely reliable as the evidence was derived from six high-quality and stringently designed RCTs. Acupuncture alone or combined with standard care also demonstrated superiority in terms of improved sleep quality (decreased PSQI score), menopausal symptoms (decreased KI score) and negative emotions (decreased HAMD and HAMA scores) compared with standard care. According to the subgroup analysis of treatment duration, it suggested that extending treatment to more than 8 weeks did not confer additional benefit. It is related to the “after-effect” of acupuncture treatment. Continuous acupuncture may lead to “fatigue” of acupuncture points, which greatly reduces the efficacy of acupuncture.

Nevertheless, quality of evidence supporting these positive results was very low to moderate owing to the risk of bias within the included RCTs and the high heterogeneities. The most frequent AE of acupuncture was bleeding and/or pain at acupoints, which usually resolved quickly after the needles were removed. Overall, acupuncture is safe in the management of menopausal insomnia, while its efficacy cannot be definitively concluded due to insufficient numbers or lack of high-quality evidence.

### 4.2. Comparison with other reviews

To the best of our knowledge, it was the first comprehensive review addressing the effectiveness of acupuncture which was an independent or adjuvant therapy in the management of menopausal insomnia. Acupuncture for menopausal insomnia was found to be more effective than sham acupuncture, standard care, or no treatment at all. Unlike previous systematic reviews [14–16], we evaluated the effects of acupuncture compared with sham acupuncture on menopausal insomnia. Sham acupuncture helps prevent bias in evaluating the specific outcome of acupuncture. In addition, our review applied stricter inclusion and exclusion criteria. Acupuncture treatment (EA or MA) was distinguished from other forms of treatment, such as auricular acupressure, acupoint catgut embedding, intradermal acupuncture and moxibustion, thereby augmenting the accuracy of the findings. Furthermore, this review first investigated the effects of acupuncture on objective sleep parameters in menopausal women with insomnia. It showed the trends between the PSQI score and objective sleep parameters were largely consistent, albeit no correlation analysis was executed. But only four included RCTs [23–25,27] investigated the effects of acupuncture on sleep structure through PSG or actigraphy, and it provided very limited evidence. We also evaluated the durability of acupunctural benefits over the follow-up period as well as the effects of the length treatment period. The subgroup analysis of our review showed that it has no obviously difference between acupuncture and western medication in the treatment duration >8 weeks, which was not mentioned in the previous reviews. Prolong acupuncture course may not increase its efficacy. Therefore, considering the economic burden of patients, when the patients of menopausal insomnia have accepted acupuncture treatment for 8 weeks in clinical practice, they can stop acupuncture for a period of time. Follow- up analysis indicates that acupuncture for menopausal insomnia not only offered short-term benefits, but also had long-term benefits (at 4 weeks).

### 4.3. Implications for future RCTs

This review suggested acupuncture can be an independent or adjuvant therapy for managing menopausal insomnia, though heterogeneity in the results indicated that the outcomes of acupuncture may be variable. The majority of the included studies showed critically low quality in terms of their designs and reporting. Several key RCT elements, including the random sequence generation, allocation concealment, and blinding, were not mentioned or were incorrectly described in more than half of the articles. Thus, it is necessary to perform adequately designed studies using rigorous methods to objectively appraise the real clinical effects of acupuncture for menopausal insomnia and to provide high-grade evidence for clinical practice. The majority of the included studies only used subjective outcome measurements. The objective outcome may be better than subjective outcome measurements to confirm the conclusion less affected by subjective factors. Women in Western countries may be more willing to adopt acupuncture as adjuvant therapy to Western medication as part of a comprehensive management program. Therefore, it is necessary to include the research on whether acupuncture could reduce drug dosage and the side effects of Western pharmacotherapy in the future. The study showed that the probability of selecting these acupoints GV20, HT7, SP6, BL23 KI3, EX-HN22, LR3, PC6, CV4, and GV24 is high, which may indicate that these acupoints have some improvement effect on the treatment of menopausal insomnia, but the specific acupuncture protocol is not clear, and further investigation on the selection of acupuncture points is needed in the future.

### 4.4. Implications for clinical practice

In this review, acupuncture achieved better improvement in the PSQI score and the KI score than sham acupuncture, standard care or no treatment. The findings suggesting that women with menopausal insomnia who experience no or a limited effect of western medication, might benefit from acupuncture alone or in combination with western medication, and it may prevent medication addiction or overuse. Menopausal women often suffered from emotional problems, which can result in the high incidence of sleep and emotional disorders. Compared with western medication, acupuncture had a great advantage in menopausal insomnia patients with negative emotions. Most of the studies involved a treatment duration of ≥4 weeks, which suggested that a treatment duration of at least 4 weeks is necessary and can serve as a reference point for the onset of action in clinical treatment, aiding clinicians in refining treatment plans and assessing efficacy. Our finding demonstrated acupuncture has better effect than sham acupuncture in sleep quality. However, it has high heterogeneity. The various methods of sham acupuncture might be the source of heterogeneity. Sham acupuncture in our studies consisted of non-insertion [23,24] or needle insertion at non-acupoints [25–27], o7r needle insertion at acupoints unrelated to menopausal insomnia [28]. Nevertheless, these methods had some limitations. Firstly, in terms of the choice of acupoints, it is difficult to find a non-effective surface point. Secondly, regarding the choice of needle insertion methods, either the superficial insertion or non-insertion all evoke activity in cutaneous afferent nerves. Therefore, it is necessary to have a consensus regarding what constitutes a high-quality sham control/placebo in future randomized sham-controlled trials, implementing defined optimal sham acupuncture in future randomized sham-controlled trials.

### 4.5. Limitations

There are several limitations in this review that need to be considered. First, the number of articles and study participants were limited, which may lead to inaccurate results. Second, given the lack of RCTs with long-term follow-up (6 months to 1 year), it is important to interpret the results cautiously. Third, blinding of both patients and investigators was difficult to achieve in many studies, increasing the risk of bias. However, in recent years, unblinded pragmatic trials have been recommended to provide clinically relevant results because of their emphasis on the practical applicability and extrapolation to real-world situations rather than treatment efficacy [51]. This design is particularly appropriate for researching complex and flexible interventions, such as acupuncture. It has also been suggested that pragmatic trials can provide more informative evidence for developing clinical guidelines for acupuncture [52,53]. Nevertheless, a gap remains between acupuncture research and its flexible application in clinical practice [54]. Finally, heterogeneity in acupuncture studies remained after subgroup analysis. Because most acupuncture methods follow the theory of traditional Chinese medicine (TCM), which emphasizes individualized treatment, the selection of acupoints and needle manipulation may have varied, inducing variation in the reported results.

## 5. Conclusion

This systematic review and meta-analysis revealed that acupuncture as an independent therapy or as an adjuvant to western medication may play a positive role in managing menopausal insomnia and is associated with few side effects, although the evidence level was low or moderate.

## Supporting information

S1 Fig Subgroup analysis for acupuncture vs. sham acupuncture according to acupuncture method. (DOCX)

S2 Fig Subgroup analysis for acupuncture vs. sham acupuncture according to treatment duration. (DOCX)

S3 Fig Subgroup analysis for acupuncture vs. western medication according to acupuncture method. (DOCX)

S4 Fig Subgroup analysis for acupuncture vs. western medication according to treatment duration. (DOCX)

S5 Fig Sensitivity analysis of PSQI scores for acupuncture vs. western medication. (DOCX)

S6 Fig Subgroup analysis for acupuncture as an adjuvant to western medication vs. western medication according to treatment duration. (DOCX)

S1 File PRISMA 2020 checklist. (DOCX)

S2 File Search strategy. (DOCX)

S1 Table Grading of evidence. (DOCX)

S1 Raw data (RAR)
