# Supplementary material for: Acupuncture as an independent or adjuvant therapy to standard management for menopausal insomnia: A systematic review and meta-analysis
Source: PLoS One. 2025 Feb 6;20(2):e0318562. doi: 10.1371/journal.pone.0318562 (PMC11801557; doi:10.1371/journal.pone.0318562)

**Supplemental Fig 2. Subgroup analysis of PSQI scores for acupuncture vs. sham acupuncture according to treatment duration (≤4 weeks or 8 weeks).**


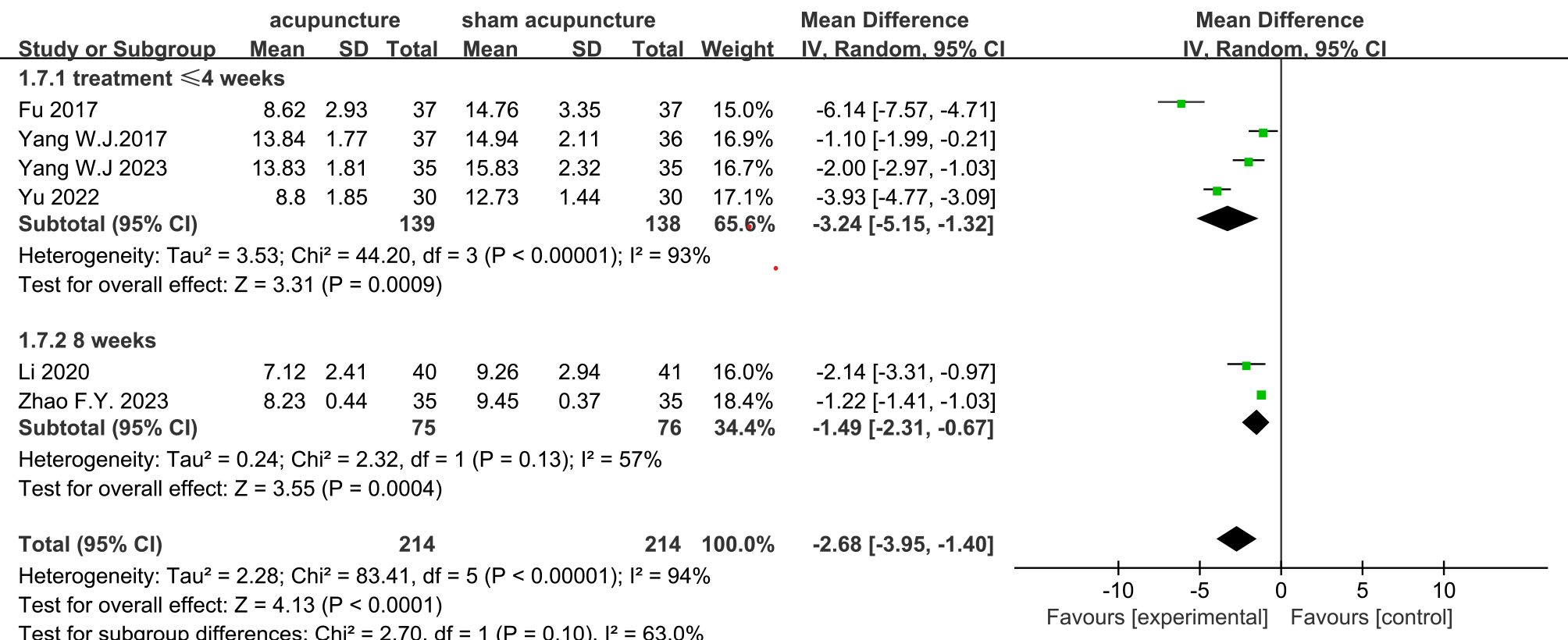

Supplement: S2 Fig — (DOCX) [file pone.0318562.s002.docx]
