# Supplementary material for: Acupuncture as an independent or adjuvant therapy to standard management for menopausal insomnia: A systematic review and meta-analysis
Source: PLoS One. 2025 Feb 6;20(2):e0318562. doi: 10.1371/journal.pone.0318562 (PMC11801557; doi:10.1371/journal.pone.0318562)
Supplement: S2 File — (DOCX) [file pone.0318562.s008.docx]

**1. Search strategies used for different databases**

**1.1 PubMed**

| No | Search items |
| --- | --- |
| 1 | "Perimenopause"[MeSH Terms] |
| 2 | "menopause"[Title/Abstract] OR "climacteric"[Title/Abstract] |
| 3 | 1 or 2 |
| 4 | "Sleep Initiation and Maintenance Disorders"[MeSH Terms] |
| 5 | "Insomnia"[Title/Abstract] OR "sleep initiation dysfunction"[Title/Abstract] OR "sleeplessness"[Title/Abstract] OR "sleep disorder"[Title/Abstract] |
| 6 | 4 or 5 |
| 7 | "acupuncture"[MeSH Terms] OR "acupuncture therapy"[MeSH Terms] |
| 8 | "acupuncture"[Title/Abstract] OR "electroacupuncture"[Title/Abstract] OR "manual acupuncture"[Title/Abstract] OR "auricular acupuncture"[Title/Abstract] OR "acupuncture point"[Title/Abstract] OR "acupoint"[Title/Abstract] OR "Electro-acupuncture"[Title/Abstract] |
| 9 | 7 or 8 |
| 10 | "randomized controlled trial"[Publication Type] OR "randomized"[Title/Abstract] OR "placebo"[Title/Abstract] |
| 11 | 3 and 6 and 9 |

**1.2 CNKI**

 ( ( ( ( ( ( 主题%='围绝经期' or 题名%='围绝经期' or title='围绝经期' or v_subject='围绝经期' ) OR ( 主题%='更年期' or 题名%='更年期' or title='更年期' or v_subject='更年期' ) ) OR ( 主题%='绝经期' or 题名%='绝经期' or title='绝经期' or v_subject='绝经期' ) ) AND ( ( 主题%='失眠' or 题名%='失眠' or title='失眠' or v_subject='失眠' ) OR ( 主题%='睡眠障碍' or 题名%='睡眠障碍' or title='睡眠障碍' or v_subject='睡眠障碍' ) ) ) AND ( ( ( ( ( 主题%='针刺' or 题名%='针刺' or title='针刺' or v_subject='针刺' ) OR ( 主题%='电针' or 题名%='电针' or title='电针' or v_subject='电针' ) ) OR ( 主题%='针灸' or 题名%='针灸' or title='针灸' or v_subject='针灸' ) ) OR ( 主题%='手捻针' or 题名%='手捻针' or title='手捻针' or v_subject='手捻针' ) ) OR ( 主题%='针法' or 题名%='针法' or title='针法' or v_subject='针法' ) ) ) AND ( ( ( 摘要='随机对照' or abstract='随机对照' ) OR ( 摘要='随机分配' or abstract='随机分配' ) ) OR ( 摘要='随机' or abstract='随机' ) ) )
