# Supplementary material for: Acupuncture as an independent or adjuvant therapy to standard management for menopausal insomnia: A systematic review and meta-analysis
Source: PLoS One. 2025 Feb 6;20(2):e0318562. doi: 10.1371/journal.pone.0318562 (PMC11801557; doi:10.1371/journal.pone.0318562)
Supplement: S1 Table — (DOCX) [file pone.0318562.s009.docx]

**Summary of findings table from GRADE profiler**

**1. acupuncture vs. sham acupuncture**

| **Quality assessment** | | | | | | | **No of patients** | | **Effect** | | **Quality** | **Importance** |  |
| --- | --- | --- | --- | --- | --- | --- | --- | --- | --- | --- | --- | --- | --- |
|  |  |  |  |  |  |  |  |  |  |  |  |  |  |
| **No of studies** | **Design** | **Risk of bias** | **Inconsistency** | **Indirectness** | **Imprecision** | **Other considerations** | **Acupuncture** | **Sham acupuncture** | **Relative (95% CI)** | **Absolute** |  |  |  |
| **PSQI (Better indicated by lower values)** | | | | | | | | | | | | |  |
| 6 | randomised trials | no serious risk of bias | serious | no serious indirectness | no serious imprecision | none | 214 | 214 | - | MD 2.68 lower (3.98 to 1.38 lower) | ⊕⊕⊕O MODERATE |  |  |
| **TST (Better indicated by lower values)** | | | | | | | | | | | | |  |
| 4 | randomised trials | no serious risk of bias | serious^1^ | no serious indirectness | no serious imprecision | none | 149 | 149 | - | MD 57.17 higher (43.29 to 71.05 higher) | ⊕⊕⊕O MODERATE |  |  |
| **SE (Better indicated by lower values)** | | | | | | | | | | | | |  |
| 4 | randomised trials | no serious risk of bias | serious^1^ | no serious indirectness | no serious imprecision | none | 149 | 149 | - | MD 11 higher (5.73 to 16.26 higher) | ⊕⊕⊕O MODERATE |  |  |
| **WASO (Better indicated by lower values)** | | | | | | | | | | | | |  |
| 4 | randomised trials | no serious risk of bias | serious^1^ | no serious indirectness | no serious imprecision | none | 149 | 149 | - | MD 38.8 lower (57.39 to 20.2 lower) | ⊕⊕⊕O MODERATE |  |  |
| **4-week follow up (Better indicated by lower values)** | | | | | | | | | | | | |  |
| 3 | randomised trials | no serious risk of bias | serious^1^ | no serious indirectness | no serious imprecision | none | 77 | 78 | - | MD 4.05 lower (7.49 to 0.61 lower) | ⊕⊕⊕O MODERATE |  |  |

^1^ The high heterogeneity

**2. acupuncture vs. western medicine**

| **Quality assessment** | | | | | | | **No of patients** | | **Effect** | | **Quality** | **Importance** |  |
| --- | --- | --- | --- | --- | --- | --- | --- | --- | --- | --- | --- | --- | --- |
|  |  |  |  |  |  |  |  |  |  |  |  |  |  |
| **No of studies** | **Design** | **Risk of bias** | **Inconsistency** | **Indirectness** | **Imprecision** | **Other considerations** | **Acupuncture** | **Western medicine** | **Relative (95% CI)** | **Absolute** |  |  |  |
| **PSQI (Better indicated by lower values)** | | | | | | | | | | | | |  |
| 16 | randomised trials | very serious^1,2^ | serious^3^ | no serious indirectness | no serious imprecision | none | 600 | 603 | - | MD 2.41 lower (3.17 to 1.65 lower) | ⊕OOO VERY LOW |  |  |
| **KI (Better indicated by lower values)** | | | | | | | | | | | | |  |
| 5 | randomised trials | very serious^1,2^ | serious^3^ | no serious indirectness | no serious imprecision | none | 154 | 153 | - | MD 5.59 lower (9.8 to 1.38 lower) | ⊕OOO VERY LOW |  |  |
| **HAMD (Better indicated by lower values)** | | | | | | | | | | | | |  |
| 3 | randomised trials | very serious^1,2^ | no serious inconsistency | no serious indirectness | no serious imprecision | none | 106 | 105 | - | MD 4.5 lower (6.01 to 3 lower) | ⊕⊕OO LOW |  |  |
| **HAMA (Better indicated by lower values)** | | | | | | | | | | | | |  |
| 3 | randomised trials | very serious^1,2^ | serious^3^ | no serious indirectness | no serious imprecision | none | 110 | 108 | - | MD 3.21 lower (5.3 to 1.11 lower) | ⊕OOO VERY LOW |  |  |

^1^ No detailed description of random methods or allccation of hidden methods
^2^ Blind method not describled in detail
^3^ The high heterogeneity

**3. acupuncture plus western medicine vs. western medicine**

| **Quality assessment** | | | | | | | **No of patients** | | **Effect** | | **Quality** | **Importance** |  |
| --- | --- | --- | --- | --- | --- | --- | --- | --- | --- | --- | --- | --- | --- |
|  |  |  |  |  |  |  |  |  |  |  |  |  |  |
| **No of studies** | **Design** | **Risk of bias** | **Inconsistency** | **Indirectness** | **Imprecision** | **Other considerations** | **Acupunture+western medicine** | **Western medicine** | **Relative (95% CI)** | **Absolute** |  |  |  |
| **PSQI (Better indicated by lower values)** | | | | | | | | | | | | |  |
| 5 | randomised trials | very serious^1,2^ | serious^3^ | no serious indirectness | no serious imprecision | none | 184 | 183 | - | MD 3.75 lower (5.34 to 2.15 lower) | ⊕OOO VERY LOW |  |  |

^1^ Blind method not describled in detail
^2^ No detailed description of random methods or allccation of hidden methods3the high heterogeneity
^3^ The high heterogeneity

**4. acupuncture vs.** **waitlist control**

| **Quality assessment** | | | | | | | **No of patients** | | **Effect** | | **Quality** | **Importance** |  |
| --- | --- | --- | --- | --- | --- | --- | --- | --- | --- | --- | --- | --- | --- |
|  |  |  |  |  |  |  |  |  |  |  |  |  |  |
| **No of studies** | **Design** | **Risk of bias** | **Inconsistency** | **Indirectness** | **Imprecision** | **Other considerations** | **Acupuncture** | **Waitlist control** | **Relative (95% CI)** | **Absolute** |  |  |  |
| **PSQI** | | | | | | | | | | | | |  |
| 1 | randomised trials | serious^1^ | no serious inconsistency | no serious indirectness | no serious imprecision | none | - | - | - | - | ⊕⊕⊕O MODERATE |  |  |
|  |  |  |  |  |  |  |  | 0% |  | - |  |  |  |

^1^ No explanation was provided
